# CMV Infection and Lymphopenia: Warning Markers of *Pneumocystis* Pneumonia in Kidney Transplant Recipients

**DOI:** 10.3389/ti.2024.12192

**Published:** 2024-01-24

**Authors:** Isabelle Eberl, Christine Binquet, Adrien Guilloteau, Mathieu Legendre, Frederic Dalle, Lionel Piroth, Claire Tinel, Mathieu Blot

**Affiliations:** ^1^ Department of Infectious Diseases, Dijon-Bourgogne University Hospital, Dijon, France; ^2^ CHU Dijon-Bourgogne, INSERM, Université de Bourgogne, CIC 1432, Module Épidémiologie Clinique, Dijon, France; ^3^ LabEx LipSTIC, University of Burgundy, Dijon, France; ^4^ Côte d´Or Haematological Malignancy Registry (RHEMCO), Dijon-Bourgogne University Hospital, Dijon, France; ^5^ Department Nephrology and Kidney Transplantation, Dijon-Bourgogne University Hospital, Dijon, France; ^6^ Department of Parasitology-Mycology, Dijon Bourgogne University Hospital, Dijon, France; ^7^ UMR PAM Université de Bourgogne Franche-Comté (UBFC), AgroSup Dijon, Équipe Vin, Aliment, Microbiologie, Stress, Groupe Interactions Candida-muqueuses, Dijon, France; ^8^ Université Bourgogne Franche-Comté (UBFC), EFS BFC, Inserm UMR1098, RIGHT, Besançon, France; ^9^ Lipness Team, INSERM Research Centre LNC-UMR1231 and LabEx LipSTIC, University of Burgundy, Dijon, France

**Keywords:** kidney transplantation, pneumonia, lymphopenia, pneumocystis, CMV

## Abstract

*Pneumocystis* pneumonia (PcP) remains life-threatening in kidney transplant recipients (KTR). Our study investigated risk factors one-year before PcP. We conducted a monocentric, case-control study including all KTR at the Dijon University Hospital (France) with a diagnosis of PcP between 2005 and 2022 (cases), and matched control KTR with no history of PcP (3 controls/case). Among all 1,135 KTR, 57 cases (5%) and 169 matched-controls were included. PcP was associated with 18% mortality. Compared to controls, cases were older, with a higher immunological risk, and CMV infection was more frequent in the year preceding the occurrence of PcP (23% vs. 4%; *p* < 0.001). As early as 1 year before PcP, lymphocyte counts were lower and serum creatinine levels were higher in cases, but immunosuppressive regimens were not significantly different. Multivariable analysis identified lymphocyte count, serum creatinine level, being treated by immunosuppressive therapy other than anti-rejection drugs, and CMV infection in the year preceding the time PcP as independently associated with the occurrence of PcP. PcP was associated with an increased risk of subsequent chronic rejection (27% vs. 3%; *p* = 0.001) and return to dialysis (20% vs. 3%; *p* = 0.002). The occurrence of CMV infection and a low lymphocyte count could redefine the indications for continuation or reinitiation of anti-*Pneumocystis* prophylaxis.

## Graphical Abstract

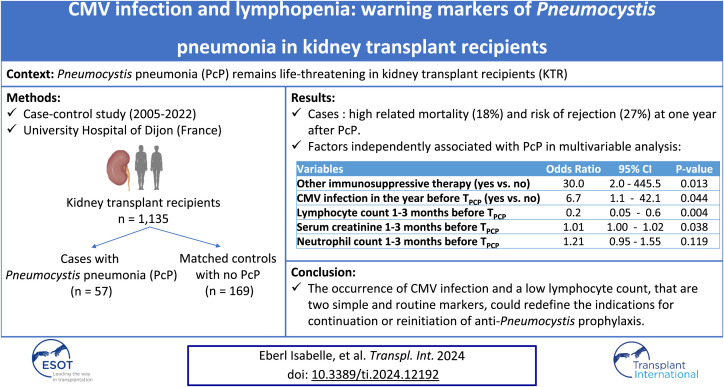

## Introduction

Infections are the third leading cause of death following kidney transplantation [1], and *Pneumocystis* pneumonia (PcP) is one of the most severe opportunistic causes. *Pneumocystis* infects 0.3%–2.6% of kidney transplant recipients (KTR), with a mortality rate reaching 14% in patients admitted to the ICU [2] and an increased risk of transplant loss in surviving patients [3]. The Kidney Disease Improving Global Outcomes (KDIGO) guidelines recommend universal initial PcP prophylaxis with cotrimoxazole for the first 3–6 months after kidney transplantation [4], while the American Society of Transplantation recommends prophylaxis for 6–12 months [5]. However, whether this prophylaxis should be prolonged or resumed in certain high risk situations remains unclear [5]. These recommendations have changed the epidemiology, and now most reports involve late post-transplant recipient PcP [6–9].

*Pneumocystis* infection elicits T-cell mediated responses including T helper (Th) 1, Th2 and Th17 responses [10], and lymphopenia has been frequently reported as an independent risk factor for PcP [6, 11–13]. Kaminski et al proposed targeted prophylaxis based on simple criteria such as chronic lymphopenia (i.e., < 1,000/μL) [6]. However, the factors that contribute to lymphopenia are not fully understood. Some studies showed that cumulative immunosuppression, corticosteroids pulses, or treated transplant rejection episodes are independent risk factors for PcP, with some conflicting results depending on the cohorts [3, 6, 11–19]. In addition, most study data are collected more than 1 year before the PCP. Identifying clinical and biological biomarkers in the year preceding the PcP could help guide clinicians regarding PcP prophylaxis.

Thus, the main objective of our study was to identify risk factors associated with PcP after kidney transplantation, with a particular focus on events occurring in the year prior PcP. The secondary objective was to study how PcP affects kidney transplant and patient outcomes.

## Material and Methods

### Study Design

We conducted a retrospective, case-control study at the University Hospital of Dijon (France) (1,200 beds). We included all KTR aged 18 years or older, with post-transplant PcP diagnosed between 2005 and 2022 (cases) and 3 matched-control KTR with no history of PcP during their follow-up (controls). Control patients were matched on the date of the active transplantation (±6 months) and selected if they had a functioning transplant at the time of PcP and with a minimal 1-year follow-up after the date of the matched PCP case. We used a simple matching strategy, using the date of transplantation to ensure a homogeneous clinical care and an equal distribution of exposure among cases and controls. Further matching variable candidates (such as induction therapy or lymphopenia) were not retained as the association of matched variables with outcomes cannot be examined. We defined T_PcP_ as the day of the microbiological confirmation of the PcP for each case and as the reference matched day from active transplantation for the matched control.

The criteria for PcP were (i) clinical signs of pneumonia (at least 2 signs among cough, sputum, chest pain, dyspnea, temperature >37.8°C or <36°C, crackles), and (ii) lung infiltration on chest x-ray or CT-scan, and (iii) a positive result on *Pneumocystis jirovecii* real-time polymerase chain reaction (PCR) testing [MycoGENIE^®^ P. jirovecii Kit ADEMTECH, Bordeaux, France] or direct immunofluorescence testing, or direct examination (Gomori-Grocott and May-Grünwald-Giemsa staining) of respiratory microbiological samples (sputum, tracheal aspirate, broncho-alveolar lavage fluid (BALF)). A diagnosis of PcP was not retained in case of a more likely diagnosis and if the curative treatment for PcP was not pursued.

First, we identified cases with the International Classification of Diseases (ICD)-10 codes in the French hospital discharge database using codes associated with kidney transplantation (Z940) and *Pneumocystis* infection (B59). These data were cross-referenced with those of the Nephrology Department of the Dijon University Hospital to identify potential missing cases. The accuracy of the diagnosis was checked in individual medical files by a trained clinician and patients were not included if they did not meet the inclusion criteria. If a patient presented several episodes of PcP, only the first was considered.

### Data Collection

Data from cases and controls were collected from medical records. A high immunological risk was defined as >1 allograft transplantation and/or positive anti-human leukocyte antigen (HLA) antibodies (before or on the day of transplantation). Cytomegalovirus (CMV) infection was defined as a positive whole blood CMV quantitative nucleic acid testing for patients from 2005 or as a positive CMV antigenemia (CMV-pp65 antigen) before that date, in accordance with the evolving diagnostic strategy in our center.

In the year before T_PCP_, at several time points (6 months-1 year, 3–6 months, 1–3 months before T_PcP_, and at T_PcP_), we collected biological data, immunosuppressive regimen including mycophenolate mofetil (MMF) and azathioprine doses, and trough levels [T_0_] for cyclosporine, tacrolimus, and mammalian target of rapamycin inhibitors (mTORi), and occurrence of infections. Clinical and radiological signs, and treatments received for PcP were collected for each case. One year after T_PcP_, we collected immune status and renal function (serum creatinine levels and estimated glomerular filtration rate [eGFR] according to the chronic kidney disease-epidemiology collaboration [CKD-EPI]). We defined allograft failure as return to permanent dialysis. Each transplant rejection was histologically proven by allograft biopsy and immunohistological examination according to the Banff classification.

### Immunosuppressive Regimen and Scoring Therapy-Related Immunosuppression

Immunosuppressive therapy strategy in our center is detailed in the Supplemental Methods.

We used a modified version of the score by Vasudev et al [20] to quantify the impact of immunosuppressive therapies, using the concept of an immunosuppression unit and based on the drug trough level (T0) instead of drug doses (Supplemental Methods). We also established the TIS (Total ImmunoSuppression) score, to take into account immunosuppressive therapies other than maintenance treatment, including corticosteroid pulses, chemotherapy treatment for solid cancer or hematological disease received in the year before T_PCP_ (Supplemental Methods). In our center, patients were treated with post-transplantation prophylaxis (oral cotrimoxazole or atovaquone if intolerance) but the duration of treatment was left to the physician’s discretion.

### Ethics

The study protocol and data collection are in accordance with French (Information Technology and Freedom Law n°78-17 of 6 January 1978) and European (GRPD EU 2016/679) good practice recommendations on data protection and patient information (Commitment of compliance MR004 n°2210228 of 3 December 2018), with written patient consent not being required for this non-interventional study. All personnel involved in organ donation and transplantation at the University Hospital of Dijon commit to respect the objectives, principles and recommendations of the Istanbul Declaration against organ trafficking and tourism in organ transplantation.

### Statistical Analysis

Quantitative values were expressed by their medians and interquartile ranges (IQR), and qualitative variables by their level’s size and percentages. Initial univariable analyses were performed using a conditional logistic regression on all available patient characteristics. In order to identify the variables independently associated with PcP, a conditional logistic regression was estimated with all the variables associated with the occurrence of PcP with a *p*-value <0.2 in univariable analysis and then a backward selection was performed using AIC. Patients with missing data were excluded. The log-linearity hypothesis for continuous variable was assessed by comparing two models, with and without the adjunction of a quadratic term, using the Likelihood Ratio Test (LRT). Results were expressed as odds ratios (OR) with 95% confidence intervals (95%CI). Stacked bar charts were plotted to represent the distribution of cases and controls according to the lymphocyte count and the occurrence of a CMV infection within the year before the time of *Pneumocystis* pneumonia. A *p*-value <0.05 was considered statistically significant. Analyses were performed using R (v4.1.3) and GraphPad Prism (v.9.1.1) software.

## Results

### Demographic and Clinical Characteristics of the Study Population

Among 1,135 kidney transplant patients, 57 patients (5%) developed PcP after transplantation between 2005 and 2022, and were considered as cases. They were matched with 169 control renal transplant patients with no history of PcP. Not all cases could be matched to 3 controls (Supplementary Figure S1). Following active transplantation, PcP occurred after a median time of 40 months (IQR 13–92) and, after prophylaxis discontinuation if applicable, a median time of 18 months (4–34).

Cases were significantly older than controls at T_PcP_ but the sex ratio and comorbidity profile did not differ between groups (Table 1). Cases had a higher immunological risk, but induction and maintenance therapies were comparable. However, cases had received significantly more other adjuvant immunosuppressive therapies prior to their active transplantation (i.e., anti-CD20 or anti-CD3 antibodies or plasma exchange). Anti-*Pneumocystis* prophylaxis was administered in half of the patients (including 100% of patients transplanted after 2007), with no difference between the two groups, but with a shorter prescription in cases compared to controls (6.0 (4.6–8.4) vs. 8.2 (5.3–15.8) months; *p* = 0.027). Acute rejection was reported in 14% of patients, with no significant difference between the two groups.

**TABLE 1:** Baseline characteristics of cases and controls.

	Missing	Controls	Cases	*p*-value
	Data	*n* = 169	*n* = 57	
**Demographic data**				
Age (years) at T_PCP_, median (IQR)	0	57 (43–65)	61 (57–66)	0.043
Male sex, n (%)	0	70 (41)	28 (49)	0.282
**Comorbidities at T_PCP_**				
Chronic heart disease, n (%)	0	36 (21)	17 (30)	0.197
Diabetes, n (%)	0	36 (21)	16 (28)	0.272
Chronic pulmonary disease, n (%)	0	6 (4)	3 (5)	0.566
Chronic liver disease, n (%)	0	12 (7)	2 (4)	0.43
Solid tumor, n (%)	0	26 (15)	10 (18)	0.751
Hematological cancer, n (%)	0	2 (1)	2 (4)	0.272
Cancer chemotherapy within a year before T_PCP_, n (%)	0	2 (1)	2 (4)	0.272
Primary underlying nephropathy	0			0.909
- Vascular, n (%)		11 (7)	3 (5)	
- Tubulo-interstitial, n (%)		27 (16)	4 (7)	
- Glomerular, n (%)		60 (36)	26 (46)	
- Polycystic kidney, n (%)		43 (25)	13 (23)	
- Others, n (%)		10 (6)	3 (5)	
- Unknown, n (%)		18 (11)	8 (14)	
Transplant data				
First transplant, n (%)	0	144 (85)	49 (86)	0.913
Age (years) at active transplant, median (IQR)	0	51 (38–60)	56 (47–60)	0.04
Living donor transplant, n (%)	2	21 (12)	5 (9)	0.469
High immunological risk, n (%)	6	70 (42)	31 (60)	0.029
Anti-HLA antibodies	9			0.107
- Transitional, n (%)		13 (8)	9 (18)	
- Constant, n (%)		52 (32)	20 (40)	
Anti-HLA antibodies at the time of transplantation, n (%)	9	56 (34)	23 (46)	0.125
Antibodies to the donor at the time of transplantation, n (%)	9	1 (1)	0	NA
CMV Status D+/R-	0	47 (28)	12 (21)	0.403
Induction therapy				
Polyclonal antibodies, n (%)	2	114 (67)	37 (69)	0.851
Anti-IL2-R, n (%)	3	50 (30)	16 (30)	1
Other induction therapy, n (%)	4	3 (2)	5 (9)	0.018
Initial immunosuppressive regimen				
Corticosteroids, n (%)	1	169 (100)	55 (98)	0.561
Calcineurin inhibitors, n (%)	1	156 (92)	49 (88)	0.234
- Ciclosporin, n (%)	1	119 (70)	34 (61)	0.208
- Tacrolimus, n (%)	1	38 (22)	14 (25)	0.703
Antimetabolites, n (%)	1	168 (99)	54 (96)	0.24
- Azathioprine, n (%)	1	12 (7)	3 (5)	0.885
- Mycophenolic acid, n (%)	1	156 (92)	51 (91)	1
m-TOR inhibitors, n (%)	1	4 (2)	4 (7)	0.097
Prophylaxis against PCP				
Cotrimoxazole, n (%)	0	81 (48)	26 (48)	0.696
Atovaquone, n (%)		2 (1)	0 (0)	1
Prophylaxis duration (month), median (IQR)		8.2 (5.3–15.8)	6.0 (4.6–8.4)	0.027
Infectious and immunological complications before T_PCP_				
Acute rejection, n (%)	1	23 (14)	8 (14)	0.482
Acute rejection in the year before T_PCP_, n (%)	1	3 (2)	2 (4)	0.448
CMV infection, n (%)	1	26 (15)	21 (37)	<0.001
CMV infection in the year before T_PCP_, n (%),	1	7 (4)	13 (23)	<0.001
Other infection, n (%)	7	41 (24)	21 (37)	
- Bacteriemia, n (%)		7 (4)	2 (4)	0.848
- Urinary tract infection (including pyelonephritis), n (%)		37 (22)	19 (33)	0.298
- Respiratory infection, n (%)		3 (2)	2 (4)	0.448
- Other infection in the year before T_PCP_, n (%)		8 (5)	4 (7)	0.42
Immunosuppressive regimen at T_PCP_, n (%)				
Corticosteroids, n (%)	0	161 (95)	57 (100)	0.208
Ciclosporin, n (%)	0	76 (45)	25 (44)	0.936
Tacrolimus, n (%)	0	52 (31)	16 (28)	0.717
m-TOR inhibitors, n (%)	0	22 (13)	14 (25)	0.041
Azathioprine, n (%)	0	14 (8)	8 (14)	0.262
Mycophenolic acid, n (%)	0	143 (85)	43 (77)	0.144
Corticosteroid pulses in the year before T_PCP_, n (%)	0	5 (3)	4 (7)	0.177
Other immunosuppressive therapy^a^, n (%)	0	3 (2)	5 (9)	0.025
Immunosuppression score				
Modified Vasudev total score, median (IQR)	0	5 (4–7)	5 (4–6.5)	0.822
TIS score, median (IQR)	0	22.5 (17.5–27.5)	25 (20–27.5)	0.17
Biological findings 1–3 months before TPCP				
Leukocytes (/mm³), median (IQR)	17	6.1 (4.7–7.4)	5.7 (4.4–8.1)	0.407
Neutrophils (/mm³), median (IQR)	20	4.2 (3.2–5.1)	3.9 (3.1–5.7)	0.032
Lymphocytes (/mm³), median (IQR)	19	1.1 (0.7–1.6)	0.7 (0.4–1)	0.001
Monocytes (/mm³), median (IQR)	20	0.6 (0.4–0.7)	0.5 (0.4–0.6)	0.137
Serum creatinine (µmol/L), median (IQR)	13	128 (103–155)	175 (133–225)	0.001
Calcemia (mmol/L), median (IQR)	18	2.4 (2.3–2.5)	2.4 (2.2–2.5)	0.536

^a^ Plasma exchanges (*n* = 2), anti-CD20 (*n* = 2), Sirolimus as anti-cancer therapy (*n* = 1), cyclophosphamide (*n* = 1), OKT3 (*n* = 1), azathioprine for an ulcerative colitis (*n* = 1).

Abbreviations: CMV, Cytomegalovirus; IQR, Interquartile range; HLA, Human Leukocyte Antigen; mTOR, Mammalian Target of Rapamycin; PCP, Pneumocystis pneumonia; T_PCP_, Time of PCP; TIS, total immunosuppression score.

**FIGURE 1:**
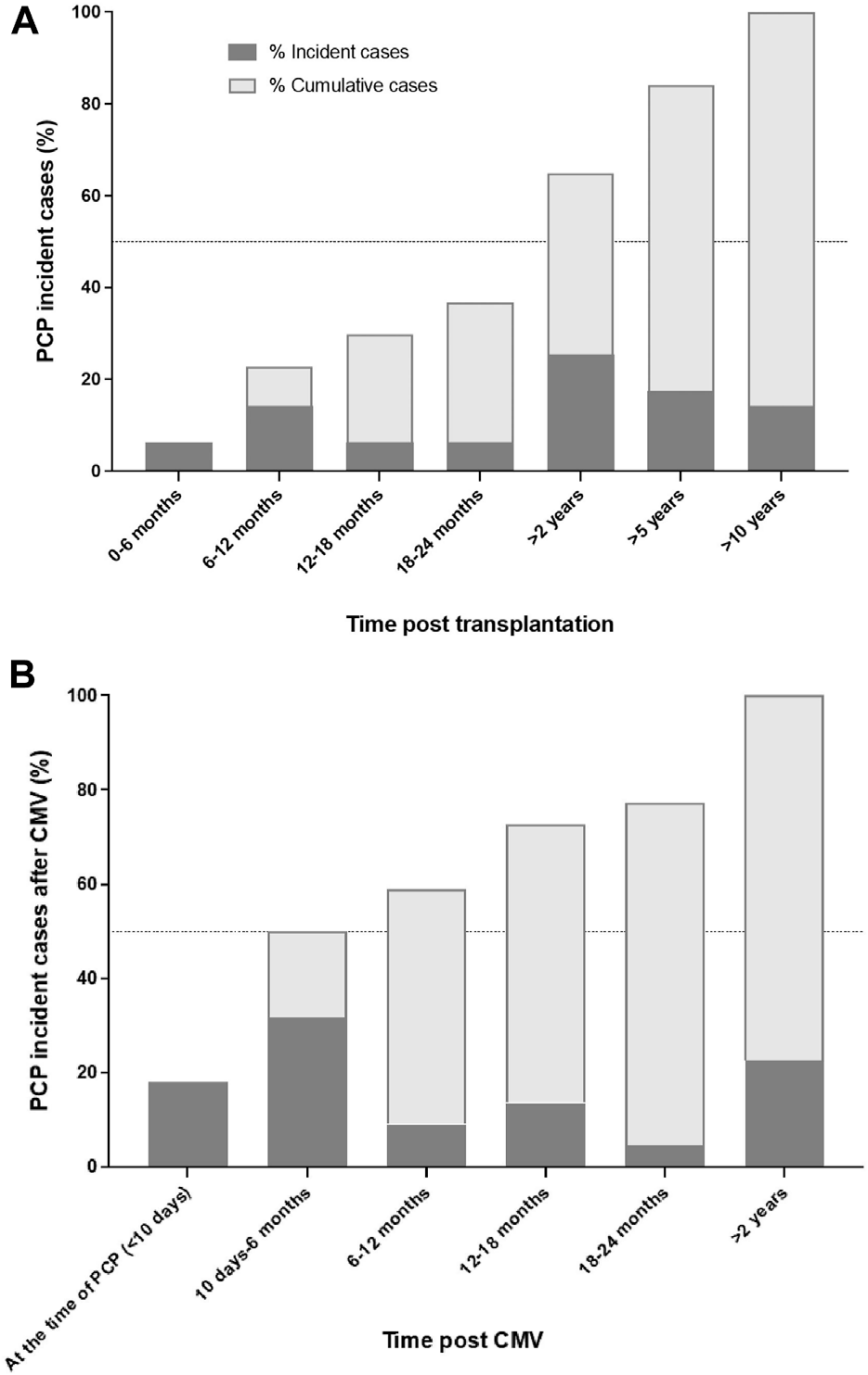
Proportion of incident and cumulative *Pneumocystis* pneumonia cases according time post transplantation **(A)** and among cases with CMV infection in the year before *Pneumocystis* pneumonia **(B)**. Abbreviations: CMV, cytomegalovirus; PCP, *Pneumocystis* pneumonia.

Cases were significantly more likely to present CMV infection than controls (37% vs. 15%; *p* < 0.001), mainly in the year before T_PcP_ (23% vs. 4%; *p* < 0.001). Among cases with a CMV infection, 17/22 (77%) developed PcP in the 2 years following the infection (Figure 1B).

**FIGURE 2:**
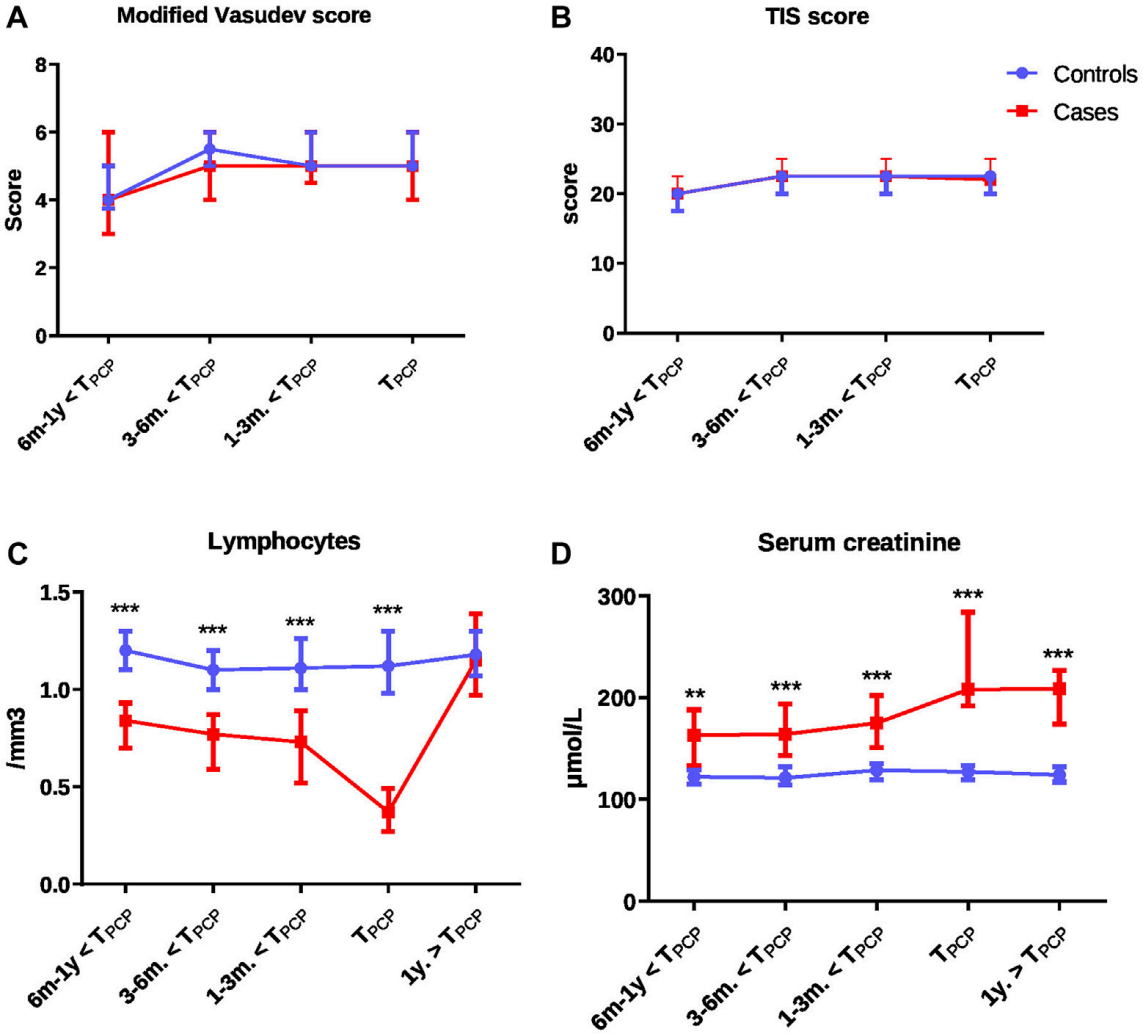
Comparison of immunosuppression scores and biological values over time from 1 year before to 1 year after the time of PCP between cases and controls: modified Vasudev score **(A)**, Therapeutic Immunosuppression (TIS) score **(B)**, lymphocytes **(C)** and serum creatinine levels **(D)**. Comparisons were performed with Wilcoxon test for matched data for each time-point with false discovery rate to correct for multiple comparisons. All panels: *p < 0.05 to <0.01; **p < 0.01 to <0.001; ***p < 0.001. Abbreviations: TIS score, therapeutic immunosuppression score; PCP, *Pneumocystis* pneumonia; T_PCP_, time of PCP.

The immunosuppressive regimen that was being administered at T_PcP_ did not differ between cases and controls, with the exception of m-Tor inhibitors, which were significantly more prescribed for cases. However, both immunosuppression scores TIS and modified Vasudev total scores did not significantly differ between cases and control at T_PcP_ and in the year before (Table 1; Figures 2A, B; Supplementary Table S1).

Lymphocyte counts were significantly lower and neutrophil counts and creatinine levels higher in cases compared to controls (Table 1). The differences in lymphocyte count and creatinine levels were present as early as 1 year before T_PCP_ (Figure 2; Supplementary Table S2).

### Factors Independently Associated With the Occurrence of PcP

**FIGURE 3:**
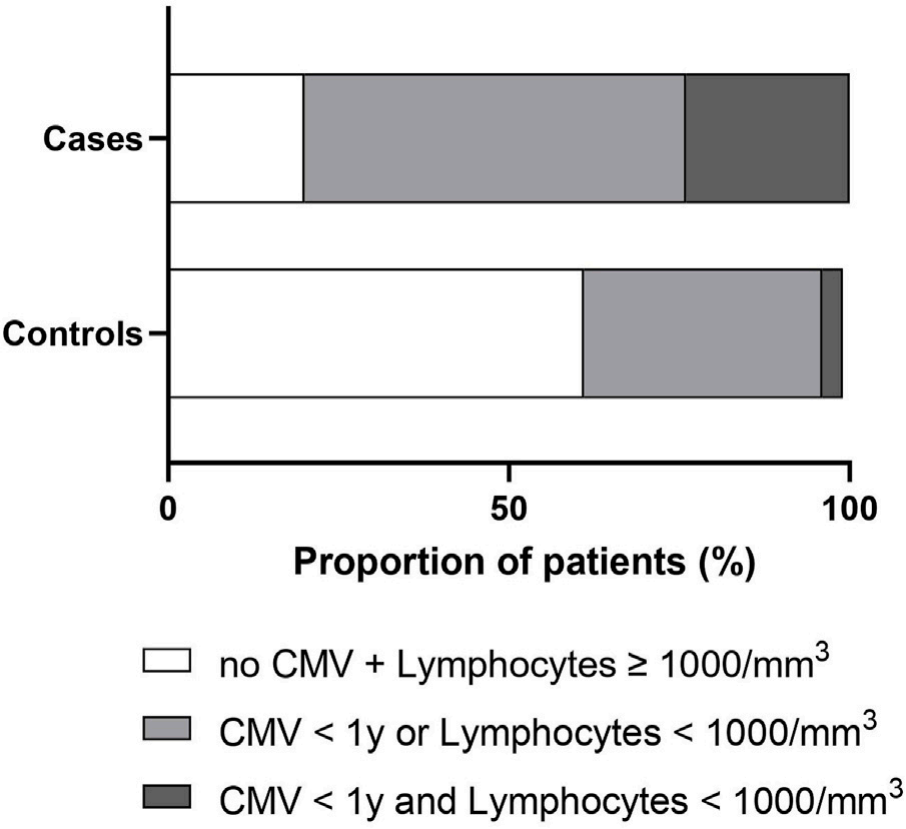
Stacked bar charts showing the distribution of cases and controls according to the lymphocyte count and the occurrence of a CMV infection within the year before the time of *Pneumocystis* pneumonia (whole population *n* = 226). Cases and controls were represented as having no CMV infection within the year and lymphocytes count ≥1,000/mm^3^ (white), CMV infection within the year or lymphocyte count <1,000/mm^3^ (light gray), and CMV infection within the year and lymphocytes count <1,000/mm^3^ (dark gray). Lymphocyte count was measured 3 months before the time of *Pneumocystis* pneumonia. Abbreviations: CMV, cytomegalovirus.

Due to missing data mainly on biological findings, the multivariable model was estimated on 44 cases and 157 controls. It showed that factors independently associated with PcP were: being treated by immunosuppressive therapy other than anti-rejection drugs, CMV infection in the year before T_PcP_, lymphocyte count and creatinine levels 1–3 months before T_PcP_ (Table 2). Thus we observed that 24% of cases had a lymphocyte count <1,000/mm^3^ and CMV infection in the year before T_PcP_, compare with only 3% of control patients (Figure 3; Supplementary Table S4). In a sensitivity analysis in patients who systematically received anti-*Pneumocystis* prophylaxis after renal transplantation (*n* = 104), we observed that 32% of cases had a lymphocyte count <1,000/mm^3^ and a CMV infection in the year before T_PcP_, compared with only 4% of control patients (Supplementary Table S5). No deviation from the hypothesis of log-linearity was identified for continuous variable (age at T_PcP_, neutrophils, lymphocytes, serum creatinine).

**TABLE 2:** Multivariable logistic regression analysis for factors associated with Pneumocystis pneumonia.

Variables	Odds ratio	95% CI	*p*-value
Other immunosuppressive therapy (yes vs. no)	30.006	2.021–445.451	0.013
CMV infection in the year before T_PCP_ (yes vs. no)	6.663	1.054–42.121	0.044
Lymphocyte count 1–3 months before T_PCP_	0.174	0.054–0.563	0.004
Serum creatinine 1–3 months before T_PCP_	1.009	1.000–1.017	0.038
Neutrophil count 1–3 months before T_PCP_	1.214	0.951–1.549	0.119

Abbreviations: PCP, Pneumocystis pneumonia; T_PCP_, Time of PCP.

### Outcomes Following PCP

At 1 year after T_PcP_, we observed 12 (21%) deaths, including 10 (18%) related to PcP in cases and no deaths in control patients. In surviving patients, cases were more likely to have high creatinine levels, transplant rejection and return to dialysis 1 year after T_PcP_ (Table 3).

**TABLE 3:** Univariable comparisons of patient outcomes at 1 year after *Pneumocystis* pneumonia.

	Missing	Controls	Cases	
	Data	*n* = 169	*n* = 57	*p-*value
Mortality, n (%)	0	0	12 (21)	<0.001
PCP-related mortality, n (%)	0	0	10 (18)	<0.001
Leukocyte count (/mm^3^), median (IQR)	32	6 (5.1–7.5)	5.8 (4.9–8.1)	0.352
Neutrophil count (/mm^3^), median (IQR)	33	4 (3.2–5)	3.7 (3–5.4)	0.703
Lymphocyte count (/mm^3^), median (IQR)	32	1.2 (0.9–1.6)	1.2 (0.8–1.5)	0.483
Monocyte count (/mm^3^), median (IQR)	34	0.6 (0.5–0.7)	0.6 (0.5–0.8)	0.807
Serum creatinine levels (µmol/L), median (IQR)	28	124 (105–159)	209 (146–252)	0.001
GFR (mL/min)^a^, median (IQR)	32	54 (38–70)	27 (23–42)	0.001
Proteinuria^b^ (g/g), median (IQR)	46	0.28 (0.17–0.51)	0.48 (0.2–1.2)	0.486
Transplant rejection, n (%)	22	5 (3)	12 (27)	0.001
Transplant rejection with need for dialysis, n (%)	21	5 (3)	9 (20)	0.002

^a^ According o the CKD-EPI, formula.

^b^ Proteinur/creatinuria ratio = Uprot [mg/L] x 8,84/Ucreat [µmol/L]) Abbreviations: GFR, Glomerular filtration rate; IQR, Interquartile range; T_PCP_, Time of *Pneumocystis* pneumonia.

## Discussion

Our case-control study involving KTR yielded 2 main results. First, PcP occurred in 5% of KTR followed in our center and was associated with high related mortality (18%), an increased risk of subsequent chronic rejection, and a return to dialysis. Secondly, several factors were independently associated with PcP, including being treated by immunosuppressive therapy other than anti-rejection drugs, CMV infection in the year before T_PcP_, low lymphocyte count, and high creatinine levels. Having a lymphocyte count <1,000/mm^3^ and/or a CMV infection are two main factors associated with the occurrence of PcP within the year.

PcP is an opportunistic infection that occurs in patients suffering from CD4^+^ T cell response deficiency, which is the case in KTR, who are thus eligible for PcP prophylaxis [4]. In our cohort, 5% developed PcP, which is within the range reported in other cohorts [2, 6, 16, 19]. However, the epidemiology has changed over the last 30 years as a result of updated recommendations and the systematic use of cotrimoxazole, leading to an increase in the proportion of late-onset PcP. It should be noted that PcP occurred in the median time of 40 months, i.e., well after the end of the theoretical prophylaxis recommendation. In this cohort, only half of patients, particularly the most recently included patients, received early prophylaxis with cotrimoxazole. By matching cases and controls on the date of the active transplantation, it is therefore not possible to study the effect of the prophylaxis variable (presence/absence) on the occurrence of PcP. However, the duration of prophylaxis was shorter for cases, suggesting that extending or reinitiating PcP prophylaxis could benefit some patients.

To identify such patients, several associated/risk factors for susceptibility to PcP have been previously identified, but with some discrepancies between studies [3, 6, 11–19]. In addition, events occurring during the year preceding PcP could be informative. As expected, cases were older than controls at the time of PcP, with frailty conferring a higher age-related risk of infection [21]. Cases were also more likely to have a higher creatinine level preceding PcP, supporting the concept of kidney impairment-associated immunosenescence [22]. They were more often considered as having a high immunological risk, raising the possibility of more likely transplant rejection. However, the proportion of acute rejection was similar in cases and controls (14% in each group).

We observed that CMV infection was independently associated with PcP, mainly in the year preceding T_PcP_. This association has been reported in several studies [14–16], but not all [6]. In the meta-analysis by Hosseini-Moghaddam et al., CMV infection significantly increased the risk of post-transplant PcP (OR: 3.30, 95% CI: 2.07–5.26). In addition, Lee et al. showed that PcP and CMV co-infection is associated with an increased clinical severity and worse clinical outcomes [23]. The causal link between CMV infection and the occurrence of PcP cannot be asserted, but pathophysiological assumptions can be proposed. First, stronger immunosuppression could be responsible for both opportunistic infections. We observed that cases were more likely to have a low lymphocyte count, as described in other work [6, 12, 13, 18]. The intensity of cumulative immunosuppression remains a difficult variable to quantify. However, we observed no significant difference in the choice of anti-rejection molecules or in the intensity of therapeutic immunosuppression, as assessed by modified Vasudev and TIS scores. Only mTOR inhibitors were more prescribed in cases compared to matched controls, as previously reported [6, 24]. As we discuss above, this association can be explained by the immunosuppressive effect of mTOR inhibitors but without ruling out the possibility of having included mTOR inhibitor-induced interstitial lung disease in some cases [24]. Furthermore, the administration of steroid pulses were not significantly associated with PcP, unlike in the study by Kaminski et al. [6, 13, 25]. However, this result should be interpreted in the light of a low frequency of acute rejection in the cohort. Other immunosuppressive therapies were more frequently prescribed in cases, mainly anti-cancer chemotherapy or anti-C5 therapies, highlighting the role of the cumulative immunosuppressive burden between transplantation and PcP. Secondly, cases were more likely to have impaired renal function, even when adjusted for age. This poorer renal function may reflect the altered terrain in which opportunistic infection occurs more frequently, as the incidence of infections increases linearly as renal function deteriorates [26]. Finally, we observed that for 3⁄4 of patients, PcP occurred within 2 years after CMV infection. CMV infection by itself can induce cellular immunodepression, through mobilization of cellular T immune defenses and secondary immunoparalysis. This hypothesis is reinforced by the results of an *in vivo* study in mice inoculated with CMV and *Pneumocystis*, showing that CMV infection induces a decrease in lung cells expressing MHC class II, and in activated T-CD4 lymphocytes in lymphoid organs and the alveolar compartment, associated with a defect in *Pneumocystis* clearance [27].

Our study confirms that PcP is associated with a poor prognosis in KTR [2, 28, 29], with an attributable mortality rate of 18% and transplant loss in 20% of surviving patients. It is therefore crucial to better understand the risk factors associated with this infection in order to define at risk-situations where anti-*Pneumocystis* prophylaxis is highly recommended. Global management of PcP involves several nephrotoxic interventions (high dose cotrimoxazole, contrast agent…) and the tapering of immunosuppressive regimen that may further elicit chronic rejection, contribute to the decline in transplant function and precipitate the return to dialysis.

In the end, we identified simple and routine biomarkers (serum creatinine, lymphocyte count) and a frequent opportunistic infectious event (CMV infection) that were associated with the occurrence of PcP. Among cases who received initial anti-*Pneumocystis* prophylaxis, 22 of 25 (88%) infections could have been prevented if prophylaxis had been restarted or continued in the presence of CMV infection and/or lymphopenia <1,000/mm^3^. This strategy would have been associated with excess treatment in of 23 out of 79 controls (29%), but is supported by the excellent tolerability of such low doses in real practice, the low cost of the drug, and the good compliance of patients.

The limitations of this study are related to its retrospective and monocentric nature. Some data are missing, even if this number is very limited for most variables. It is possible that over this period of 17 years, unmeasured changes in clinical practice may have influenced the risk to contract PCP, but such difference have been minimized by the controls pairing strategy. We did not provide CD4 and CD8 lymphocytes count since lymphocyte immunophenotyping has only become part of routine follow-up in more recent years and CD4 counts are thus not available for all patients. However, in the study of Kaminsky et al. lymphopenia was identified as the most significantly associated lymphocytic marker of PCP [6]. Patient prognosis could only be partially evaluated and is potentially biased insofar as the matching imposed a follow-up time for controls that was at least equal to that of the index case plus 1 year. Some patients had not received anti-*Pneumocystis* prophylaxis, but our sensitivity analysis confirmed the same findings in the subgroup of patients who received prophylaxis.

## Conclusion

PcP is associated with high mortality and transplant loss in patients who have undergone a kidney transplant. We identified factors that were independently associated with PcP, including immunosuppressive therapy other than anti-rejection drugs, CMV infection in the year before T_PcP_, low lymphocyte count and high serum creatinine levels. These risk factors remain unchanged with or without anti-*Pneumocystis* prophylaxis. Based on these results and previous literature, the occurrence of CMV infection and/or lymphopenia <1,000/mm^3^ could redefine the indications for continuation or reinitiation of anti-*Pneumocystis* prophylaxis, which is an inexpensive and well-tolerated treatment.

## Data Availability

The raw data supporting the conclusion of this article will be made available by the authors, without undue reservation.

## Abbreviations

CMV, Cytomegalovirus; HLA, Human Leukocyte Antigen; KTR, Kidney Transplant Recipients; PcP, *Pneumocystis* pneumonia; rATG, recombinant anti-lymphocyte depleting antibodies; TIS, Total Immunosuppression.
