# Supplementary material for: CMV Infection and Lymphopenia: Warning Markers of Pneumocystis Pneumonia in Kidney Transplant Recipients
Source: Transpl Int. 2024 Jan 24;37:12192. doi: 10.3389/ti.2024.12192 (PMC10849047; doi:10.3389/ti.2024.12192)
Supplement: Supplementary file 1 [file DataSheet2.docx]

**Factors associated with *Pneumocystis* pneumonia after renal transplantation: a case-control study**

**Supplemental methods**

**Immunosuppressive regimen**

All induction therapy used at our center before 2004 consisted in anti-lymphocyte depleting antibodies (rATG, Thymoglobulin®, Sanofi-Aventis). From 2004, anti-interleukin 2 receptors alpha antibodies (anti-IL2RA, basiliximab on days 0 and 4 post-transplant or daclizumab) were preferentially used, and rATG administered only for HLA-sensitized patients (presence of anti-HLA antibodies directed or not against antigenic determinants of the graft, > 1 transplant). The initial immunosuppressive regimen at our center consists of a combination therapy with calcineurin inhibitors, either tacrolimus (target concentrations 8-12 ng/ml) or ciclosporin (target concentrations 150-200 ng/ml for the first 3 months, then 75-125 ng/ml), an antimetabolite, mainly mycophenolate mofetil (2000 mg per day and decreased thereafter), or azathioprine (2 mg/kg pr day) and steroids. Some patients received mTORi (rapamycin, target concentrations between 5 and 8 ng/L). For graft rejection, whether acute cellular or humoral, corticosteroid boluses (1g) were administered for 3 days. Thymoglobulins can be associated in case of cortico-resistant rejection. In case of humoral rejection, anti-CD20 therapy (rituximab), plasma exchange or immunoglobulin courses could be associated.

**Modified Vasudev Score**

| **Corticosteroids** | | **Azathioprine** | | **Ciclosporine** | | **Tacrolimus** | | **Mycophenolate Mofetil** | |
| --- | --- | --- | --- | --- | --- | --- | --- | --- | --- |
| Dosage* | IS Unit | Dosage* | IS Unit | T0** | IS Unit | T0** | IS Unit | Dosage* | IS Unit |
| 0 | 0 | 0-50 | 0 | 0-50 | 0 | 0-5 | 0 | 0 | 0 |
| 5 | 1 | 50-100 | 0.5 | ≥50-100 | 0.5 | ≥5-10 | 1 | 500 | 1 |
| 10 | 2 | 100-150 | 1 | ≥100-150 | 1 | ≥10 | 2 | 1000 | 2 |
| Pulse*** | 8 | 150-200 | 1.5 | ≥150-200 | 1.5 |  |  | 1500 | 3 |
|  |  | ≥ 200 | 2 | ≥200 | 2 |  |  | 2000 | 4 |

*Total daily dose of prednisone or prednisolone in mg

** Residual drug concentrations in µg/L

***corticosteroid pulse (methylpredisolone), often 3 days in a row, daily dose 500 mg

"Normal" immediate post-transplant score = 8 units

Corticosteroid bolus or chemotherapy within the year preceding T_PcP_= 8 units

**Total Immunosuppression (TIS) score**

| **Prednisone** | | **Mycophenolate mofetil** | | **Azathioprine** | | **Tacrolimus** | | **Ciclosporine** | | **mTORi** | |
| --- | --- | --- | --- | --- | --- | --- | --- | --- | --- | --- | --- |
| Dosage* | Score | Dose* | Score | Dose** | Score | T0*** | Score | T0*** | Score | T0*** | Score |
| 0 | 0 | 0 | 0 | 0 | 0 | 0 | 0 | 0 | 0 | 0 | 0 |
| 5 | 5 | 500 | 2.5 | 0.5 | 2.5 | <4 | 2.5 | <50 | 2.5 | <4 | 2.5 |
| 10 | 10 | 1000 | 5 | 1 | 5 | ≥4-6 | 5 | ≥50-100 | 5 | ≥4-6 | 5 |
| Pulse*** | 30 | 1500 | 7.5 | 1.5 | 7.5 | ≥6-8 | 7.5 | ≥100-150 | 7.5 | ≥6-8 | 7.5 |
|  |  | 2000 | 10 | 2 | 10 | ≥8 | 10 | ≥150 | 10 | ≥8 | 10 |

*Total daily dose of prednisone or prednisolone in mg

**Total daily dose in mg/kg

*** Residual drug concentrations in µg/L

**** corticosteroid pulse (methylpredisolone), often 3 days in a row, daily dose 500 mg

Chemotherapy if received within one year=30

"Normal" score in immediate post-transplant = 30 (Prednisone 10 mg/d + Mycophenolate mofetil 2g/d + Tacrolimus 8-12 µg/L)

“Maximum” score = 50 (corticosteroid bolus + Mycophenolate mofetil 2g/d + Tacrolimus 8-12 µg/L)
