# Supplementary material for: CMV Infection and Lymphopenia: Warning Markers of Pneumocystis Pneumonia in Kidney Transplant Recipients
Source: Transpl Int. 2024 Jan 24;37:12192. doi: 10.3389/ti.2024.12192 (PMC10849047; doi:10.3389/ti.2024.12192)
Supplement: Supplementary file 2 [file DataSheet1.docx]

**CMV infection and lymphopenia: warning markers of *Pneumocystis* pneumonia in kidney transplant recipients**

**Supplemental data**

**Supplemental Figure 1.** Flowchart


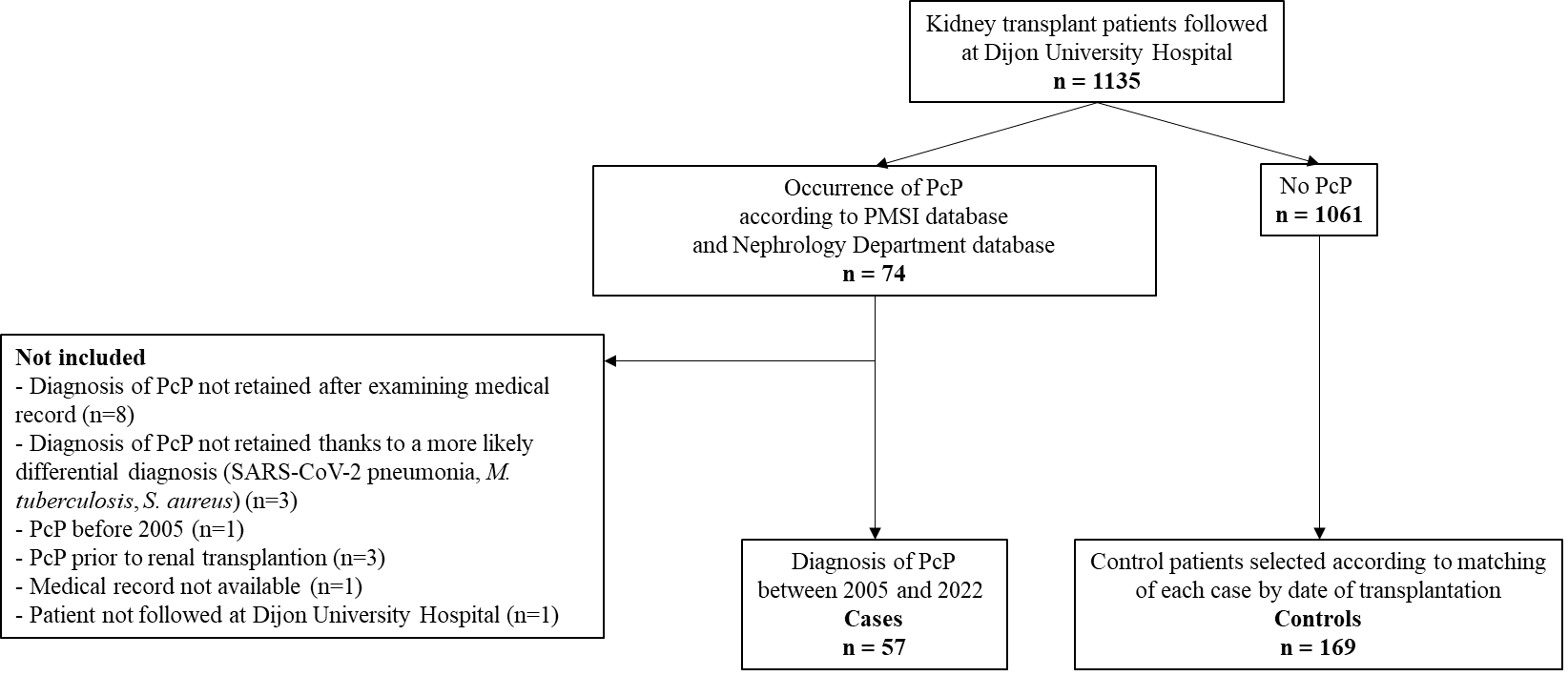


Abbreviations : PcP: *Pneumocystis* pneumonia, PMSI: Programme de Médicalisation des Systèmes d'Information

**Supplemental Table 1.** Immunosuppressive therapies in the year preceding T_PCP_

|  | **Missing** | **Controls** | **Cases** | ***P-value*** |
| --- | --- | --- | --- | --- |
|  | **Data** | **n = 169** | **n = 57** |  |
| **[1 year - 6 months[ before T_PCP_** |  |  |  |  |
| **Immunosuppressive regimen** |  |  |  |  |
| - Corticosteroids, n (%), md=53 | 53 | 123 (95) | 43 (100) | 0.268 |
| - Cyclosporin, n (%), md=53 | 53 | 59 (45) | 20 (47) | 0.843 |
| - Tacrolimus, n (%), md=53 | 53 | 36 (28) | 14 (33) | 0.542 |
| - mTOR inhibitors, n (%), md=53 | 53 | 20 (15) | 7 (16) | 0.889 |
| - Azathioprine, n (%), md=53 | 53 | 13 (10) | 5 (12) | 0.988 |
| - Mycophenolate mofetil, n (%), md=53 | 53 | 107 (82) | 33 (77) | 0.421 |
| - Other, n (%) | 53 | 1 (1) | 1 (2) | 0.997 |
| **Immunosuppression score** |  |  |  |  |
| - TIS score, median (IQR) |  | 20 (10-25) | 20 (10-27.5) | 0.263 |
| - Modified Vasudev total score, median (IQR) |  | 4 (1.75-6) | 4.5 (2-6.5) | 0.537 |
| **[6 months -3 months[ before T_PCP_** |  |  |  |  |
| **Immunosuppressive regimen** |  |  |  |  |
| - Corticosteroids, n (%) | 12 | 152 (95) | 54 (100) | 0.208 |
| - Ciclosporin, n (%) | 12 | 71 (44) | 25 (46) | 0.806 |
| - Tacrolimus, n (%) | 12 | 51 (32) | 15 (28) | 0.573 |
| - mTORi, n (%) | 12 | 21 (13) | 12 (22) | 0.109 |
| - Azathioprine, n (%) | 12 | 13 (8) | 8 (15) | 0.153 |
| - Mycophenolate mofetil, n (%) | 12 | 136 (85) | 40 (74) | 0.069 |
| - Other, n (%) |  | 0 (0) | 2 (4) | 0.103 |
| **Immunosuppression score** |  |  |  |  |
| - Modified Vasudev total score, median (IQR) |  | 5.5 (3.5-6.5) | 5 (3-6.5) | 0.639 |
| - TIS score, median (IQR) |  | 22.5 (17.5-25) | 25 (17.5-27.5) | 0.222 |
| **[3 months - 1 month[ before T_PCP_** |  |  |  |  |
| **Immunosuppressive regimen** |  |  |  |  |
| - Corticosteroids, n (%) | 0 | 161 (95) | 57 (100) | 0.208 |
| - Ciclosporin, n (%) | 0 | 76 (45) | 25 (44) | 0.884 |
| - Tacrolimus, n (%) | 0 | 52 (31) | 16 (28) | 0.701 |
| - mTORi, n (%) | 0 | 22 (13) | 14 (25) | 0.039 |
| - Azathioprine, n (%) | 0 | 14 (8) | 8 (14) | 0.205 |
| - Mycophenolate mofetil, n (%) | 0 | 143 (85) | 43 (77) | 0.18 |
| - Other, n (%) | 0 | 0 (0) | 5 (9) | < 0.001 |
| **Immunosuppression score** |  |  |  |  |
| - Modified Vasudev total score, median (IQR) |  | 5 (4-7) | 5 (4-6.5) | 0.626 |
| - TIS score, median (IQR) |  | 22.5 (17.5-27.5) | 25 (20-27.5) | 0.159 |
| **In the year before T_PCP_** |  |  |  |  |
| Corticosteroid pulses in the year before T_PCP,_ n (%) | 0 | 5 (3) | 4 (7) | 0.335 |
| **At the T_PCP_** |  |  |  |  |
| **Immunosuppressive regimen** |  |  |  |  |
| - Corticosteroids, n (%) | 0 | 160 (95) | 57 (100) | 0.166 |
| - Corticosteroid pulse in the year before T_PCP_, n (%) | 0 | 3 (2) | 2 (4) | 0.803 |
| - Ciclosporin, n (%) | 0 | 76 (45) | 24 (42) | 0.706 |
| - Tacrolimus, n (%) | 0 | 52 (31) | 16 (28) | 0.776 |
| - mTORi, n (%) | 0 | 23 (14) | 16 (28) | 0.012 |
| - Azathioprine, n (%) | 0 | 15 (9) | 9 (16) | 0.147 |
| - Mycophenolate mofetil, n (%) | 0 | 144 (85) | 37 (65) | < 0.001 |
| - Other, n (%) | 0 | 0 (0) | 2 (4) | 0.103 |
| - Chemotherapy in the year before T_PCP,_ n (%) | 0 | 2 (1) | 2 (4) | 0.568 |
| **Immunosuppression score** |  |  |  |  |
| - Modified Vasudev total score, median (IQR) |  | 5 (4-6.5) | 5 (3.5-6.5) | 0.584 |
| - TIS score, median (IQR) |  | 22.5 (17.5-25) | 22 (17.5-25) | 0.842 |

Abbreviations : *Pneumocystis* pneumonia, CMV : Cytomegalovirus, mTORi : mammalian target of rapamycin inhibitors, TIS: total immunosuppression score, T_PCP_: time of PCP

**Supplemental Table 2.** Biological characteristics in the year before T_PCP_

|  | **Missing** | **Controls** | **Cases** | ***P-value*** |
| --- | --- | --- | --- | --- |
|  | **Data** | **n = 169** | **n = 57** |  |
| **[1 year - 6 months[ before T_PCP_** |  |  |  |  |
| - Leukocytes (/mm³), median (IQR) | 65 | 5.9 (4.9-6.9) | 5.2 (4.6-6.2) | **0.085** |
| - PNN (/mm³), median (IQR) | 67 | 3.8 (3.2-4.8) | 3.5 (2.8-4.3) | **0.23** |
| - Lymphocytes (/mm³), median (IQR) | 66 | 1.2 (0.8-1.5) | 0.8 (0.5-1) | **< 0.001** |
| - Monocytes (/mm³), median (IQR) | 67 | 0.5 (0.4-0.6) | 0.6 (0.5-0.7) | **0.546** |
| - Serum creatinine (µmol/L), median (IQR) | 59 | 122 (100-150) | 163 (107-200) | **0.04** |
| **[6 months -3 months[ before T_PCP_** |  |  |  |  |
| - Leukocytes (/mm³), median (IQR) | 28 | 5.8 (4.7-7) | 5.4 (4.1-6.8) | **0.247** |
| - PNN (/mm³), median (IQR) | 32 | 3.9 (2.9-4.9) | 3.7 (2.7-5.3) | **0.769** |
| - Lymphocytes (/mm³), median (IQR) | 31 | 1.1 (0.8-1.6) | 0.8 (0.5-1) | **< 0.001** |
| - Monocytes (/mm³), median (IQR) | 32 | 0.6 (0.4-0.7) | 0.6 (0.4-0.7) | **0.452** |
| - Serum creatinine (µmol/L), median (IQR) | 23 | 121 (104-151) | 164 (129-210) | **< 0.001** |
| **[3 months - 1 month[ before T_PCP_** |  |  |  |  |
| - Leukocytes (/mm³), median (IQR) | 17 | 6.1 (4.7-7.4) | 5.7 (4.4-8.1) | **0.661** |
| - PNN (/mm³), median (IQR) | 20 | 4.2 (3.2-5.1) | 3.9 (3.1-5.7) | **0.845** |
| - Lymphocytes (/mm³), median (IQR) | 19 | 1.1 (0.7-1.6) | 0.7 (0.4-1) | **< 0.001** |
| - Monocytes (/mm³), median (IQR) | 20 | 0.6 (0.4-0.7) | 0.5 (0.4-0.6) | **0.126** |
| - Serum creatinine (µmol/L), median (IQR) | 13 | 128 (103-155) | 175 (133-225) | **< 0.001** |
| - Calcemia (mmol/L), median (IQR) | 18 | 2.4 (2.3-2.5) | 2.4 (2.2-2.5) | **0.984** |
| **At the T_PCP_** |  |  |  |  |
| - Leukocytes (/mm³), median (IQR) | 15 | 5.8 (5-7.3) | 5.8 (4.4-7.8) | **0.532** |
| - PNN (/mm³), median (IQR) | 20 | 4 (3.1-4.9) | 4.5 (2.8-6) | **0.248** |
| - Lymphocytes (/mm³), median (IQR) | 20 | 1.1 (0.8-1.6) | 0.4 (0.2-0.6) | **< 0.001** |
| - Monocytes (/mm³), median (IQR) | 20 | 0.6 (0.5-0.7) | 0.4 (0.3-0.6) | **< 0.001** |
| - Creatinine levels (µmol/L), median (IQR) | 11 | 127 (105-158) | 208 (154-321) | **< 0.001** |
| - Calcemia (mmol/L), median (IQR) | 18 | 2.35 (2.25-2.46) | 2.53 (2.37-2.8) | **< 0.001** |

Abbreviations : Pneumocystis pneumonia, PNN: polynuclear neutrophil, T_PCP_: time of PCP

**Supplemental Table 3.** Clinical and radiological features and therapeutics during *Pneumocystis* pneumonia

|  | **Cases** |
| --- | --- |
|  | **n = 57** |
| Time between active transplant and PCP (months), median (IQR) | 40 (13-92) |
| **Clinical signs** |  |
| Fever > 38°C, n (%) | 42 (74) |
| Cough, n (%) | 33 (58) |
| Dyspnea, n (%) | 53 (93) |
| Oxygen-requirement, n (%) | 46 (82) |
| **Radiological signs** |  |
| **Chest X-ray** |  |
| - chest X-ray performed, n (%) | 55 (96) |
| - interstitial syndrome, n (%) | 53 (96) |
| **Thoracic CT scan** |  |
| - CT scan performed, n (%) | 37 (76) |
| -- Lung abnormalities on CT scan, n (%) | 37 (100) |
| -- Ground glass patterns, n (%) | 26 (70) |
| -- Micro-nodular lesions, n (%) | 3 (8) |
| -- Consolidation, n (%) | 9 (24) |
| **Microbiological diagnosis of PCP** |  |
| - *Pneumocystis jirovecii* cysts and/or trophozoites at the microscopic examination, n (%) | 22 (39) |
| - *Pneumocystis* DNA detection, n (%) | 50 (87) |
| -- Sputum | 7 (12) |
| -- BALF | 50 (88) |
| **Co-infections** |  |
| - Bacterial coinfections | 14 (25) |
| - Viral coinfections | 32 (56) |
| - Fungal coinfections | 3 (5) |
| **Treatments during PCP** |  |
| Tappering of immunosuppressive regimen, n (%) | 39 (74) |
| Transfer to ICU, n (%) | 17 (30) |
| Dialysis, n (%) | 14 (25) |
| Mechanical ventilation, n (%) | 11 (20) |

Abbreviations: PCP: Pneumocystis pneumonia, BALF: Bronchoalveolar lavage fluid, ICU: Intensive Care Unit,

**Supplemental Table 4**. Distribution of patients (cases and controls) according to CMV infection within the year of T_PcP_ and the lymphocytes count (whole population n=226)

|  | **Controls** | **Cases** |
| --- | --- | --- |
|  | **n = 169** | **n = 57** |
| Lymphocytes <1000/mm3 | 58 (39) | 36 (80) |
| CMV infection in the year before T_PcP_ | 7 (4) | 13 (23) |
| No CMV infection in the year before T_PcP_ and lymphocytes ≥ 1000/mm3 | 91 (61) | 9 (20) |
| CMV infection in the year before T_PcP_ or lymphocytes < 1000/mm3 | 54 (35) | 25 (56) |
| CMV infection in the year before T_PcP_ and Lymphocytes < 1000/mm3 | 5 (3) | 11 (24) |

Abbreviations: *Pneumocystis* pneumonia, CMV: Cytomegalovirus, T_PCP_: time of PCP

**Supplemental Table 5.** Distribution of patients (cases and controls) according to CMV infection within the year of T_PcP_ and the lymphocytes count : sensitivity analysis on the population of patients who received initial prophylaxis and for whom data are available (n=104)

|  | **Controls** | **Cases** |
| --- | --- | --- |
|  | **n = 79** | **n = 25** |
| Lymphocytes <1000/mm3 | 23 (29) | 22 (88) |
| CMV infection in the year before T_PCP_ | 4 (5) | 8 (31) |
| No CMV infection in the year before T_PcP_ and lymphocytes ≥ 1000/mm3 | 55 (70) | 3 (12) |
| CMV infection in the year before T_PcP_ or lymphocytes < 1000/mm3 | 21 (27) | 14 (56) |
| CMV infection in the year before T_PcP_ and Lymphocytes < 1000/mm3 | 3 (4) | 8 (32) |

Abbreviations: CMV: cytomegalovirus, PcP: Pneumocystis pneumonia, T_PCP_: time of PCP
